# Heparanase: a potential marker of worse prognosis in estrogen receptor-positive breast cancer

**DOI:** 10.1038/s41523-021-00277-x

**Published:** 2021-05-28

**Authors:** Tamar Zahavi, Mali Salmon-Divon, Roberto Salgado, Michael Elkin, Esther Hermano, Ariel M. Rubinstein, Prudence A. Francis, Angelo Di Leo, Giuseppe Viale, Evandro de Azambuja, Lieveke Ameye, Christos Sotiriou, Asher Salmon, Nataly Kravchenko-Balasha, Amir Sonnenblick

**Affiliations:** 1 Tel Aviv Sourasky Medical Center, Tel Aviv, Israel; 2 Sackler Faculty of Medicine, Tel Aviv University, Tel Aviv, Israel; 3 Department of Molecular Biology, Adelson School of Medicine, Ariel University, Ariel, Israel; 4 Division of Research, Peter MacCallum Cancer Centre, Melbourne, VIC Australia; 5 Department of Pathology, GZA-ZNA Hospitals, Antwerp, Belgium; 6 Department of Oncology, Hadassah Medical Organization and Faculty of Medicine, Hebrew University of Jerusalem, Jerusalem, Israel; 7 The Institute of Biomedical and Oral Research, Hebrew University of Jerusalem, Jerusalem, Israel; 8 Peter MacCallum Cancer Centre, University of Melbourne, Melbourne, VIC Australia; 9 Breast Cancer Trials Australia & New Zealand, Newcastle, NSW Australia; 10 International Breast Cancer Study Group, Bern, Switzerland; 11 Sandro Pitigliani Department of Medical Oncology, Hospital of Prato, Prato, Italy; 12 The University of Milan, and IEO European Institute of Oncology IRCCS, Milan, Italy; 13 Institut Jules Bordet and l’Université Libre de Bruxelles (U.L.B), Brussels, Belgium; 14 Ministry of Health, Jerusalem, Israel

**Keywords:** Oncology, Cancer

## Abstract

Heparanase promotes tumor growth in breast tumors. We now evaluated heparanase protein and gene-expression status and investigated its impact on disease-free survival in order to gain better insight into the role of heparanase in ER-positive (ER+) breast cancer prognosis and to clarify its role in cell survival following chemotherapy. Using pooled analysis of gene-expression data, we found that heparanase was associated with a worse prognosis in estrogen receptor-positive (ER+) tumors (log-rank *p* < 10^−10^) and predictive to chemotherapy resistance (interaction *p* = 0.0001) but not hormonal therapy (Interaction *p* = 0.62). These results were confirmed by analysis of data from a phase III, prospective randomized trial which showed that heparanase protein expression is associated with increased risk of recurrence in ER+ breast tumors (log-rank *p* = 0.004). In vitro experiments showed that heparanase promoted tumor progression and increased cell viability via epithelial–mesenchymal transition, stemness, and anti-apoptosis pathways in luminal breast cancer. Taken together, our results demonstrated that heparanase is associated with worse outcomes and increased cell viability in ER+ BC.

## Introduction

Heparanase (HPSE) cleaves glycosaminoglycan heparan sulfate (HS), a linear polysaccharide composed of repeating units of hexuronic acid and *N*-acetylglucosamine attached to the core proteins of heparan sulfate proteoglycans^1^. HS proteoglycans are ubiquitously found both at the cell surface (syndecans 1-4 and glypicans 1-6) and in the extracellular matrix (ECM) (perlecan, collagen type XVIII, and agrin). HS chains bind to and assemble with ECM proteins, thus playing important roles in ECM integrity and cell–ECM interactions^2^. In addition, HS chains regulate the activity of a variety of bioactive molecules (cytokines and growth factors) at the cell surface and in the ECM^3^. Given this functional diversity, degradation of HS by heparanase profoundly affects a variety of pathophysiological processes, including tumorigenesis and inflammation^4–6^.

Heparanase is known to enhance the progression of many cancer types and is associated with a poor prognosis. Indeed, patients bearing tumors that express high levels of heparanase had a significantly shorter postoperative survival time^7,8^. During cancer progression, the enzymatic action of heparanase may contribute to the breakdown of extracellular barriers to cell invasion^9^, regulate the bioavailability of HS-binding growth factors (bFGF, VEGF, and HGF), create a tumor-promoting inflammatory microenvironment^2^, and generate bioactive HS fragments which potentiate growth factor-receptor binding and signaling^1,5,10–15^.

While the pro-tumorigenic properties of heparanase are well documented, little is known about its function in chemoresistance. Heparanase was reported to promotes autophagy and enhance tumor growth and chemoresistance in head and neck carcinoma^16^. It was also demonstrated that the tumor cells express a much higher level of heparanase upon relapse among patients with multiple myeloma following high-dose chemotherapy than was present prior to therapy^17^. In addition, the involvement of heparanase in myeloma resistance to drug therapy was found to be dependent upon its ability to increase stemness properties^18^.

Although the role of heparanase in tumor progression and upregulation of its abundancy have been detected in breast cancer^19–21^, the specific function of heparanase in the chemoresistance of breast cancer has not yet been explored. Our present study focuses specifically on the involvement of heparanase in chemoresistance of ER-positive (ER+) breast tumors. Most patients with ER+ breast tumors, have a good prognosis with hormone therapy alone. However, in some patients with poor prognosis, a combination of both adjuvant chemotherapy and hormone therapy may be recommended. The decisions regarding the addition of chemotherapy to adjuvant hormone therapy in ER-positive patients are individualized and take into account the benefits that are expected from therapy^22,23^.

Here, we used the findings of a large pooled analysis and a prospective clinical trial in order to examine whether heparanase is associated with the outcome in breast cancer and to establish the role of heparanase in cell survival following chemotherapy in ER+ breast cancer.

## Results

### Pooled analysis of heparanase gene expression and outcome in patients with breast cancer

**Fig. 1:**
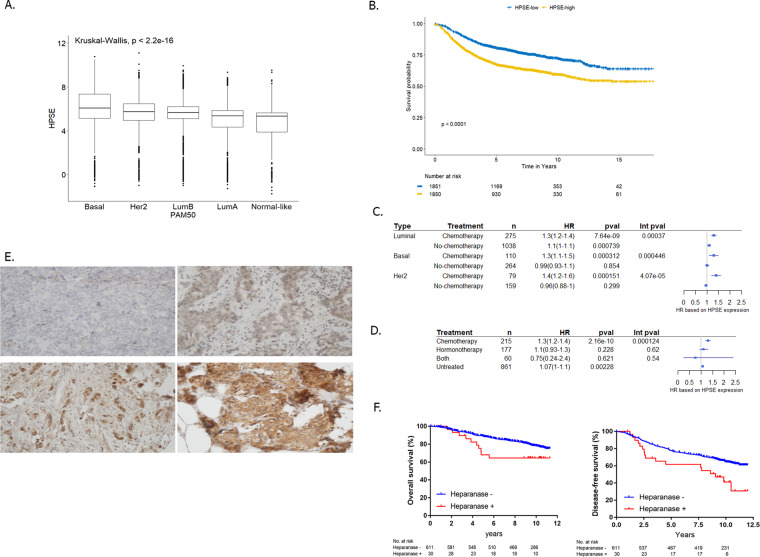
Analysis of heparanase expression and outcome in patients with breast cancer. **A** Using the PAM50 classification model, patients were assigned to the main breast cancer molecular subtypes: Luminal A (*n* = 3239), Luminal B (*n* = 3375), basal (*n* = 2074), HER2+ (*n* = 1444), and normal-like (*n* = 566). Basal and HER2-enriched cancers were more likely to possess high levels of heparanase in comparison to luminal cancers. **B** We assessed the prognostic value of quartiles of heparanase gene expression in all breast cancer patients (*n* = 3701). Significance (*p*-values) of differences in survival between patient groups as defined by quartiles of heparanase expression is estimated by the log-rank test. **C** Forest plots according to heparanase gene expression in luminal ER-positive, basal, and HER2-enriched patients. The plots indicate Cox regression hazard ratios, 95% confidence intervals, and *p*-values for chemotherapy benefit for DDFS, as well as *p*-values of the interaction (int pval) between heparanase gene expression and chemotherapy treatment. **D** Forest plots according to heparanase gene expression in luminal ER-positive patients. The plots indicate Cox regression hazard ratios, 95% confidence intervals, and *p*-values for chemotherapy or hormonal benefit for DDFS, as well as *p*-values of the interaction (int pval) between heparanase gene expression and chemotherapy or hormone therapy treatment. **E** TMAs were stained for heparanase by IHC. The staining was analyzed according to the intensity (range: 0–3) right = 0, left =3 strong staining. **F** Heparanase Kaplan–Meier survival curves in the BIG 2-98 cohort. OS = left and DFS = right.

We used publically available microarray data sets comprising over 10,000 breast cancer patients to build a pooled set of gene-expression profiles with available outcome data in order to delineate the clinical relevance of heparanase in breast cancer^24^. We employed the PAM50 classification model, and patients were assigned to one of the main breast cancer molecular subtypes, namely, Luminal A, Luminal B, HER2-enriched, basal-like, and normal-like breast cancers. We first assessed whether heparanase expression was associated with any particular subtype and observed that it was expressed significantly more in the basal and HER2 subtypes in comparison to the luminal subtypes (*p* < 0.01^−10^) (Fig. 1A). We next assessed whether heparanase was correlated with survival in breast cancer patients for whom relapse data were available. As shown in Fig. 1B, heparanase was significantly associated with worse prognosis in the general population (*p* < 0.01^−10^) and in each breast tumor subtypes separately (Supplementary Fig. 1). Next, we assessed the clinical benefit of chemotherapy according to heparanase expression. The risk of disease recurrence following chemotherapy for patients with increased heparanase expression was significantly greater than for patients with low heparanase expression, regardless of tumor molecular subtype (Fig. 1C), suggesting that heparanase expression is predictive for chemotherapy resistance. While chemotherapy is indicated in early triple-negative and HER2 positive tumors, the decisions regarding the addition of chemotherapy to adjuvant hormone therapy in ER+ patients is individualized and dependent on different parameters. We, therefore, evaluated the predictive role of heparanase on the benefit derived from chemotherapy or hormonal therapy in ER+ breast tumors. In contrast to chemotherapy, the risk of disease recurrence following hormone therapy was not dependent upon heparanase expression (Fig. 1D).

### Association of heparanase with clinic-pathologic characteristics and outcome in the BIG 2-98 randomized trial

We next aimed to confirm our observation of heparanase-dependent chemotherapy resistance in ER+ tumors at the proteomic level by analyzing prospective data from the BIG 2-98 adjuvant trial repository (Supplementary Fig. 2). There were 641 ER+ tumor TMAs available for evaluation of heparanase by IHC. Various levels (on an intensity score of 1 to 3+) of heparanase staining by IHC in tumor cells were detected in 220 out of 641 samples (35%) (Fig. 1E). Heparanase was not significantly associated with any pathological parameter except for the proliferative marker Ki-67 (*p* = 0.006) (Table 1). Since it was difficult to distinguish between background (0 score) and a low level of intensity (1+ score), it was decided to consider only a score of ≥2 as positive for further survival analysis. We examined heparanase association with disease-free survival (DFS) and overall survival (OS) endpoints. As shown in Fig. 1F, heparanase was associated with worse DFS (log-rank test *p* = 0.004; hazard ratio: 2.03; 95% CI 1.24–3.33) and showed a trend towards worse OS (log-rank test *p* = 0.059; hazard ratio: 1.84; 95% CI 0.97–3.50).

**Table 1:** Association between heparanase (by IHC) expression and pathological clinical parameters in ER+ breast cancer.

		Heparanase					
	All (*N* = 641)	Negative (*N* = 611)		Positive (*N* = 30)		*p*-value	
*Age, years*							
<50	339	53%	322	53%	17	57%	0.67
≥50	302	47%	289	47%	13	43%	
*No. of involved nodes*							
1–3	345	54%	327	54%	18	60%	0.47
4–10	212	33%	205	34%	7	23%	
>10	84	13%	79	13%	5	17%	
*Tumor size, no.*							
≤2 cm	207	33%	197	33%	10	33%	0.93
>2 cm	429	67%	409	67%	20	67%	
pTx	5		5				
*Tumor grade, no.*							
G1-G2	340	55%	322	55%	18	60%	0.59
G3	276	45%	264	45%	12	40%	
Gx	25		25				
*ER, no.*							
ER−	51	8%	49	8%	2	7%	1
ER+	585	92%	558	92%	27	93%	
Missing info	5		4		1		
*PR, no.*							
PR−	101	16%	97	16%	4	14%	1
PR+	526	84%	501	84%	25	86%	
Missing info	14		13		1		
*HER2, no.*							
HER2−	526	83%	505	84%	21	70%	0.07
HER2+	105	17%	96	16%	9	30%	
Missing info	10		10				
*KI-67, no.*							
<14	139	22%	138	23%	1	3%	0.006
≥14	480	78%	451	77%	29	97%	
Missing info	22		22				

### Heparanase effect on cell survival following chemotherapy

**Fig. 2:**
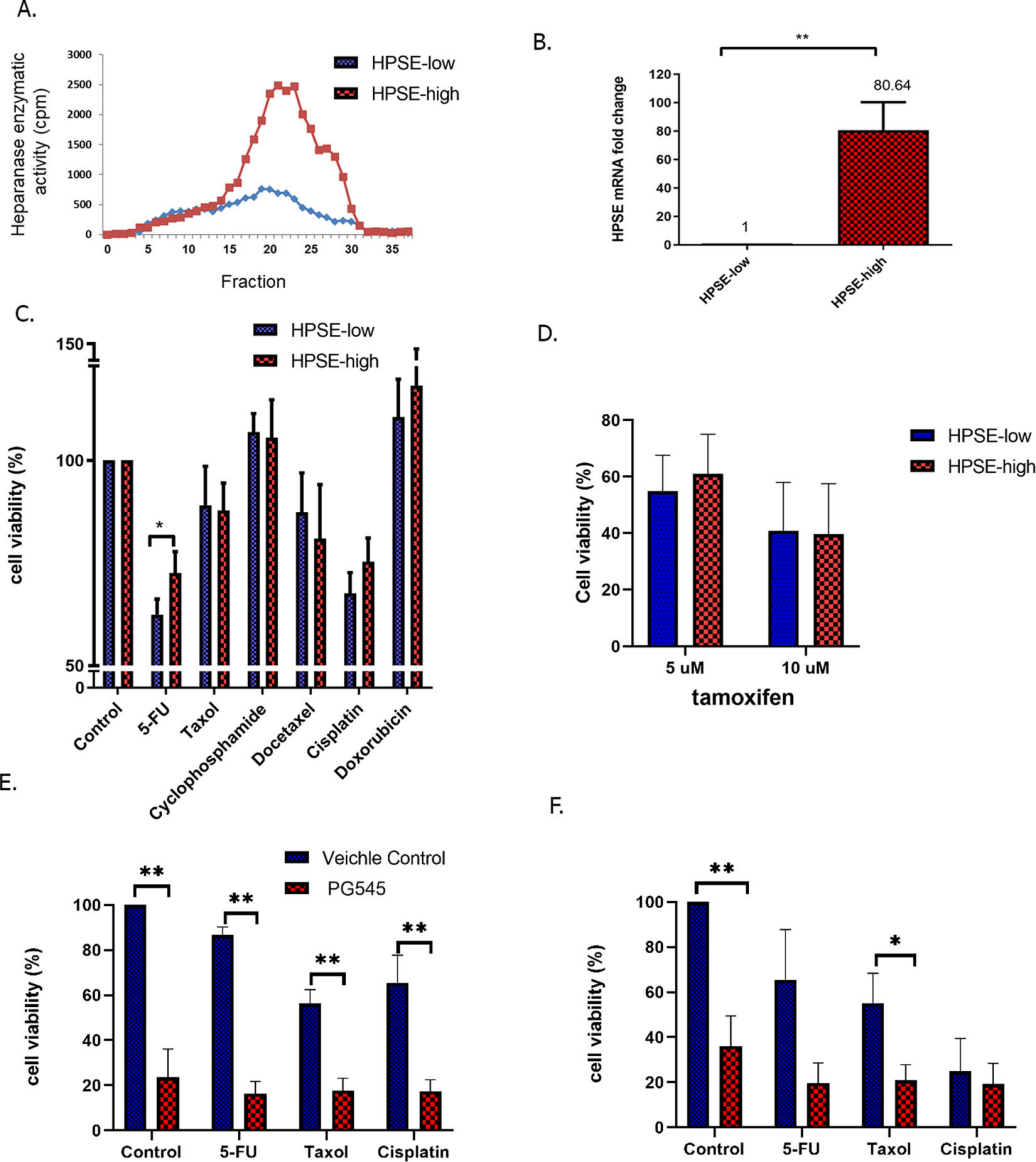
Effect of heparanase on cell viability of breast cancer cells treated with various chemotherapy agents. **A** Heparanase overexpression in HPSE-high MCF7 (as compared to control HPSE-low MCF7) cells was confirmed by activity assay. **B** Expression of heparanase in HPSE-low or HPSE-high cells, as determined by real-time PCR. **C** Cell viability MTT assay of HPSE-low or HPSE-high cells treated with the indicated chemotherapy agents for 72 h. **D** Cell viability MTT assay of HPSE-low or HPSE-high cells treated with the tamoxifen for 72 h. **E**, **F** MDA-MB-231 (**E**) and SKBR3 (**F**) breast cancer cells were treated with PG545 or vehicle (PBS only) and with different chemotherapy drugs for 48 h after which cell viability was determined by MTT assay. Cell viability was presented as the mean ± SEM of the percentage of control vs. treated cells for all MTT experiments. For all experiments: *p < 0.05, **p < 0.01.

Since heparanase was significantly associated with worse prognosis in our pooled analysis and in the BIG 2-98 trial in which all patients received adjuvant chemotherapy (including anthracycline, cyclophosphamide, methotrexate, and 5-fluorouracil ± taxane), we speculated that heparanase would affect cell viability following chemotherapy in addition to its known role in tumor aggressiveness. MCF7 (ER+) luminal breast cancer cells^12^ were treated with different chemotherapy agents, which are standard treatment in BC and had been used in the BIG 2-98 trial, and cell viability was examined by means of MTT assay. Since MCF7 cells display low levels of the heparanase enzyme^25^ we utilized the HPSE-high MCF7 cell line, which expresses a high level of heparanase (transfected with HPSE− with high enzymatic activity). We used HPSE-low MCF7 cells (transfected with empty vector) as a control (Fig. 2A). Quantitative real-time PCR revealed an over 85-fold increase in heparanase levels in the HPSE-high compared with the HPSE-low MCF7 cells (Fig. 2B). Applying the MTT assay, we found that chemotherapy affected the viability of HPSE-high cells following 5-fluorouracil (5-FU) treatment, in which HPSE-high cells demonstrated significantly higher cell viability compared to HPSE-low cells (Fig. 2C). Further estimation of cell viability using methylene blue staining confirmed these effects (Supplementary Fig. 3). In contrast, heparanase, did not affect the viability of cells treated with tamoxifen (Fig. 2D). These results support our pooled analysis, which showed that heparanase is associated with worse outcomes in ER+ breast tumors treated with adjuvant chemotherapy but not with hormone therapy.

Although our study was focused upon ER+ breast tumors, we sought to extend our in vitro experiments to other cell lines that originated from different breast cancer types. For this aim, we used MDA-MB-231 (triple-negative) and SKBR3 (HER2+) cell lines. Since these cell lines express high levels of heparanase^12^, they were treated with the heparanase inhibitor PG545^26^. As shown in Fig. 2E, F, PG545 reduced MDA-MB-231 and SKBR3 cell viability compared with vehicle control cells. Notably, no difference was seen in the cell viability following each chemotherapy treatment in comparison with the combination therapy of the chemotherapy and heparanase inhibitor. A similar lack of synergistic effect was obtained following treatment of the MDA-MB-231 cells with another heparanase inhibitor, SST0001 (also called Roneparstat) (Supplementary Fig. 4).

### Heparanase promotes cell survival following 5-fluorouracil treatment in HPSE-high MCF7 cells through distinct mechanisms

**Fig. 3:**
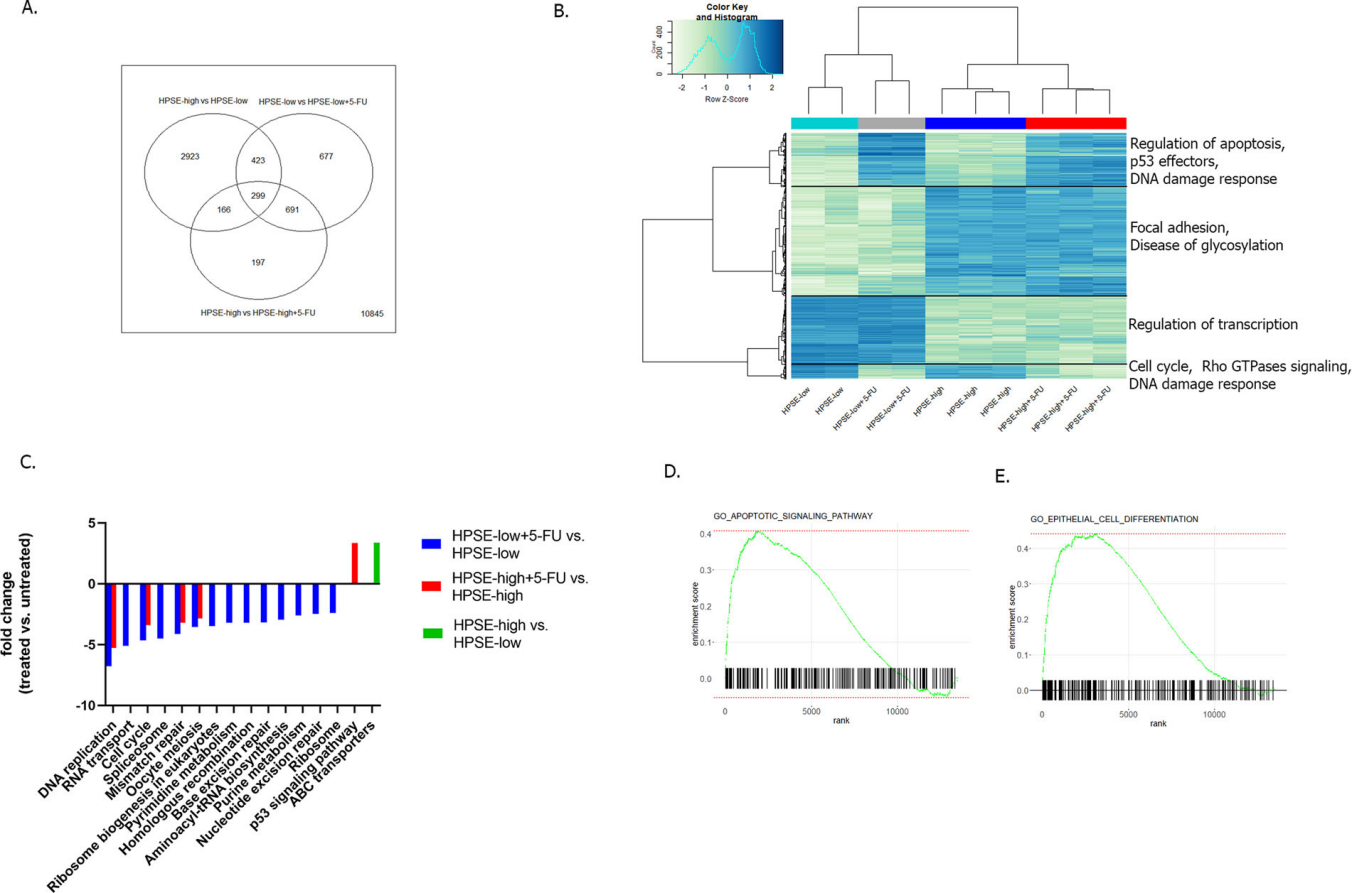
Gene-expression profile in HPSE-high MCF7 cells following treatment with 5-FU. **A** Venn diagram showing overlap of gene-expression differences associated with 5-FU treatment in HPSE-high compared with HPSE-low MCF7 cells. **B** Heat map and hierarchical clustering of the overall transcriptome differences. Gene set enrichment analysis detected differentially expressed pathways (FDR < 0.05) following treatment with 5-FU in HPSE-high and HPSE-low control cells, such as regulation of apoptosis, DNA damage response, and cell cycle (**C**) A gene set enrichment analysis. **D**, **E** Gene Set Enrichment Analysis (GSEA) enrichment plot involved in apoptosis (**D**) and epithelial cell differentiation (**E**) pathways.

A global analysis of gene expression was performed in order to understand the involvement of heparanase in cell survival following 5-FU treatment. We used an RNA-Sequencing analysis of MCF7 cells to compare differences in gene expression in HPSE-high and HPSE-low MCF7 cells with or without 5-FU treatment (Supplementary Table 1). The comparison is illustrated in a Venn diagram of gene-expression differences (Fig. 3A). To determine the expression patterns of mRNAs, a heat map was constructed to profile the overall transcriptome differences. We identified potential pathways that may be associated with the higher viability of HPSE-high cells following 5-FU treatment, including cell cycle, regulation of apoptosis, DNA damage response, etc. (Fig. 3B). Gene set enrichment analysis (using GAGE, Fig. 3C) showed that treatment with 5-FU inhibited DNA replication, cell cycle, and mismatch repair in both low and high HPSE cells, leading to the death of rapid-growing neoplastic cells. However, inhibition of those signals following 5-FU treatment was slightly more effective in the low-HPSE cells (Fig. 3C). Additional signals, such as homologous recombination, base excision repair, and pyrimidine metabolism, which promote an appropriate cell division, were down-regulated in response to 5-FU treatment only in HPSE-low cells (Fig. 3C), probably contributing to the low-survival rate of HPSE-low cancerous cells. Furthermore, enrichment analysis of known functional biological pathways (using GeneAnalytics application) with a list of DE genes with an FDR < 0.05 and log2 of fold-change ≥1.5 revealed enrichment of the apoptosis pathway in the HPSE-high but not in the HPSE-low MCF7 cells following 5-FU treatment (illustrated using GSEA, Fig. 3D). We also revealed enrichment of the epithelial cell differentiation pathway, which includes genes that are negative regulators of epithelial cell differentiation (Fig. 3E).

**Fig. 4:**
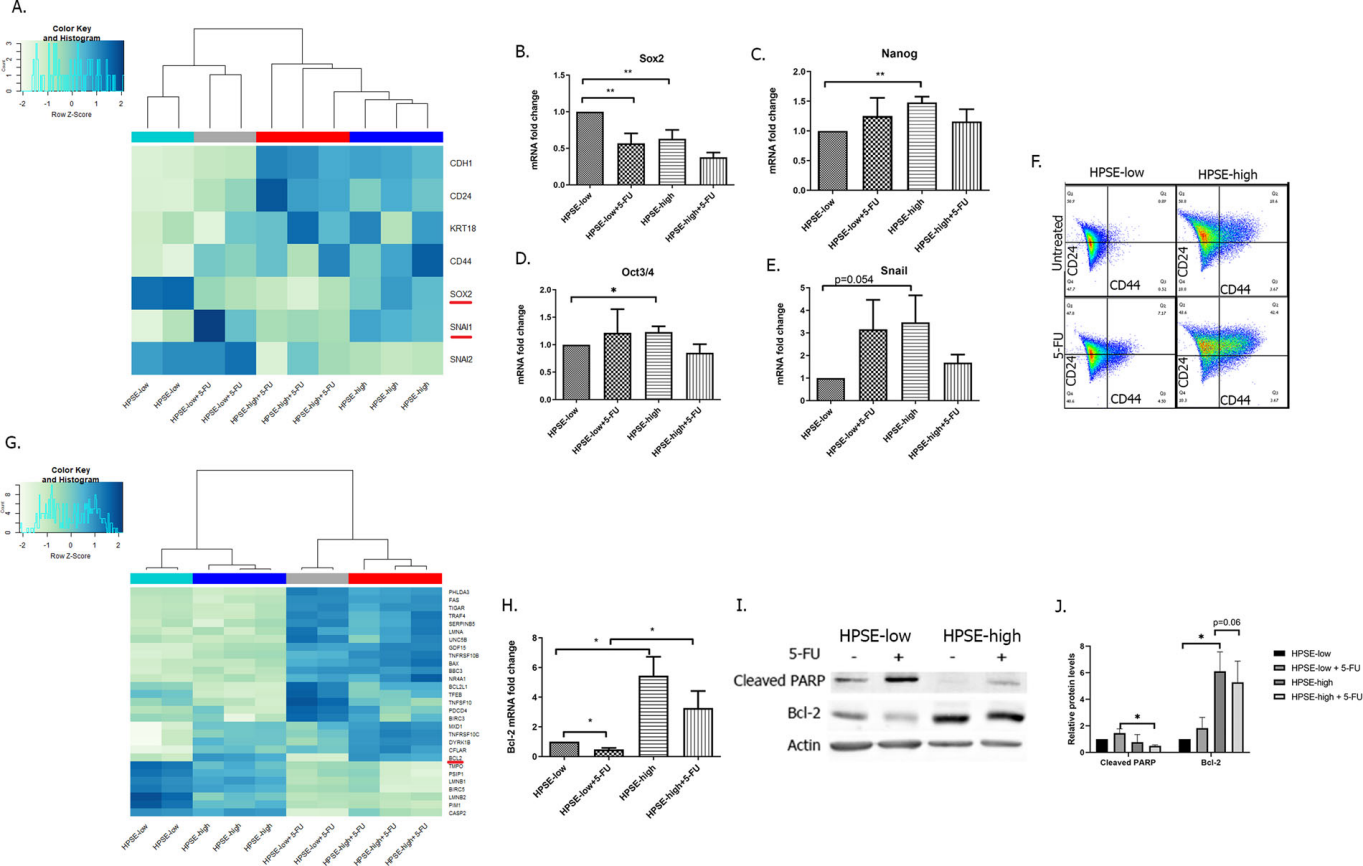
Heparanase promotes tumor progression and increases cell survival via EMT, stemness, and an anti-apoptosis pathway. **A** heat map and hierarchical clustering of stemness and EMT genes. **B**–**E** Expression of **B** Sox2, **C** Nanog, **D** Oct3/4 and **E** Snail in HPSE-low or HPSE-high cells following 5-FU treatment for 24 h, as determined by real-time PCR. **F** FACS analysis of the cell surface markers CD24 and CD44 in HPSE-low or HPSE-high following 5-FU treatment for 72 h. The provided results are from a representative experiment repeated three times. **G** heat map and hierarchical clustering of core apoptosis-related genes. **H** Expression of Bcl-2 in HPSE-low or HPSE-high cells following 5-FU treatment for 24 h, as determined by real-time PCR. **I** Western blot analysis and **J** quantification of total protein levels of Bcl-2 and cleaved PARP from HPSE-low or HPSE-high following 5-FU treatment for 24 h. All blots derive from the same experiment and were processed in parallel. For all experiments: *p < 0.05, **p < 0.01.

Since cellular plasticity is a major contributor to tumor progression and therapy failure, and our enriched analysis identified that HPSE-high is associated with regulators of differentiation we evaluated whether high HPSE expression is associated with distinguishable differentiation states. Using our RNA-Seq results, we performed a heat map and hierarchical clustering of stemness and EMT genes. The results showed significant differences in stemness and EMT expression genes between HPSE-low and HPSE-high cells, regardless of 5-FU treatment (Fig. 4A). We confirmed these results using real-time PCR expression of embryonic stem cell (ESCs) markers, such as Oct3/4, Snail, and Nanog^27^, that increased significantly in HPSE-high compared to low MCF7 cells. Although other markers (Sox2) demonstrated inverse results (Fig. 4B–E). In agreement with these observations, flow cytometry of CD44/CD24 (known cancer stem cell [CSC] markers in breast cancer^28^ and chemotherapy resistance^29^) showed that the expression level of CD44 was higher in the HPSE-high compared to the HPSE-low MCF7 cells regardless of 5-FU treatment (Fig. 4F). Notably, 5-FU treatment led to overexpression of CD44 in both HPSE-high and HPSE-low cells, however, the expression of CD44 on HPSE-high cells was even higher while CD24 expression remained constant throughout the experiment.

As mentioned above, our RNA-sequencing data revealed also a difference in the apoptosis pathway in HPSE-high, but not in HPSE-low MCF7 cells following 5-FU treatment. Heat map and hierarchical clustering of core apoptosis-related genes show a clear distinction between HPSE-low and HPSE-high cells (Fig. 4G). Among others, Bcl-2, which is a cell survival protein best known for its roles in inhibiting apoptosis^30^, was up-regulated in HPSE-high, but not in HPSE-low MCF7 cells regardless of 5-FU treatment. We validated these results by using real-time PCR (Fig. 4H). In agreement with those results, western blot detected high expression of Bcl-2 and low expression of cleaved PARP, an indicator of apoptosis, in HPSE-high MCF7 cells, regardless of 5-FU treatment. The opposite results were obtained in HPSE-low MCF7 cells (Fig. 4I, J).

Taken together, these results suggest that heparanase promotes the survival of cells, at least partially, via stemness, EMT, and anti-apoptotic pathway.

## Discussion

In this study, we found that elevated heparanase expression is associated with an increased risk of recurrence in ER+ breast tumors. The relationships between heparanase and poor prognosis have been demonstrated in various carcinomas, sarcomas, and hematologic malignancies^7,31^. In breast cancer, Sun et al.^32^ published that heparanase expression is up-regulated and associated with larger tumor size, increased lymph node metastasis, higher-grade tumor, and low-survival rates. However, the clinical data sample size was small in the latter study, and different methods were used to determine HPSE expression (real-time PCR analysis and immunohistochemistry), which may contribute to high variability in the findings of the meta-analysis. In the current study, our data sample comprised over 10,000 breast cancer patients in whom heparanase expression was determined uniformly by microarray. Therefore, thanks to the large database, we could use the PAM50 classification model and analyze the data according to subtypes. This analysis demonstrated, that heparanase is associated with worse outcomes in breast tumors. Furthermore, we found that in ER+ breast tumors the risk of disease recurrence following chemotherapy, but not hormone therapy, for patients with increased heparanase expression was greater than for patients with low heparanase expression. We also confirmed our observations at the proteomic level in another independent study, which included prospective data from the BIG 2-98 trial repository.

Heparanase is a key component of the breast tumor microenvironment and it was shown to be involved in primary breast cancer progression by creating a microenvironment that supports tumor growth, angiogenesis, and survival^5^. Various lines of evidence have shown that heparanase expression is associated with the tumorigenic potential of breast cancer^5,20,33^. However, the complete mechanism(s) exerted by heparanase to promote cancer progression is still incompletely understood in the context of breast cancer tumors. Herein, we addressed these issues by using breast cancer cells in an in vitro cell-based assay. By conducting an RNA-seq analysis, we showed that many genes were significantly differentially expressed in MCF7 breast cancer cells^12^ following heparanase overexpression. Bioinformatics analysis of these differential genes suggested that heparanase allows tumor progression through different mechanisms, including dedifferentiation, luminal progenitors regulation, and EMT induction. These results were also confirmed by real-time PCR and FACS analysis, which found a significant increase in the expression of stem cell markers, such as Oct3/4 Nanog and CD44, and in the EMT associated gene, Snail, following heparanase overexpression.

The information, which accumulated in the literature about the impact of heparanase on breast cancer chemoresistance, is limited. The results of the present study suggest that heparanase has a role in increased cell viability following chemotherapy in ER+ breast cancer cells. In support of this viewpoint, we found that HPSE-high MCF7 cells demonstrated significantly higher cell viability following treatment with 5-FU chemotherapy. Additionally, HPSE-high MCF7 cells generally responded to the chemotherapy treatment less than control HPSE-low MCF7 cells, as indicated by the lower number of differentially expressed genes and less effective inhibition of DNA replication and cell cycle signals after 5-FU treatment.

After indicating that heparanase increase ER+ tumor cell survival following chemotherapy, we sought to investigate the molecular mechanisms behind this role. The known molecular mechanisms of chemoresistance include transporter pumps, tumor suppressor genes, oncogenes, DNA repair, autophagy, mitochondrial alteration, EMT, exosome, apoptosis, and cancer stemness^34^. Here, we showed that some of these molecular mechanisms underlie the role of heparanase in increasing breast cancer cell survival. First, the ABC transporters proteins that are involved in the export of drugs from cancer cells and thereby decrease intracellular drug concentration^18^ were up-regulated only in HPSE-high MCF cells, but not in control HPSE-low MCF7 cells. Second, our results suggested that heparanase effect on cell survival is dependent upon its ability to increase stemness properties. This is in line with a previous study that revealed that heparanase involvement in the resistance of myeloma to drug therapy is dependent upon its ability to increase stemness properties in vitro^18^. Finally, we showed that heparanase increased cell survival following 5-FU treatment in MCF7 human breast cancer cells via the anti-apoptotic pathway. This finding is consistent with the known anti-apoptotic effect of heparanase in melanoma cells^35^. We assume that the different molecular mechanisms of chemoresistance, which were found to be induced by heparanase, contribute synergistically to its involvement in breast cancer survival following drug therapy.

Taken together, our results demonstrate the importance of heparanase in increasing cell viability of breast cancer and may help identify patients that can benefit from adjuvant chemotherapy in ER+ breast cancer.

## Methods

### Patients and study design

BIG 2-98 (ClinicalTrials.gov identifier of BIG 2-98: NCT00174655) is a multicenter, prospective, open-labeled, randomized phase III adjuvant trial^22^ that enrolled early and locally advanced lymph node-positive breast cancer patients who were assigned to different adjuvant chemotherapy regimens. Institutional ethics committees at all participating sites approved the study. All patients provided written informed consent prior to study entry. The patients were randomly assigned to one of four treatments in a 2 × 2 trial design as follows: Arm 1 (sequential control): (A) doxorubicin 75 mg/m^2^ × 4 every 3 weeks → classical CMF (cyclophosphamide, methotrexate and 5-fluorouracil) × 3; Arm 2 (concurrent control): (AC) doxorubicin, cyclophosphamide 60/600 mg/m^2^ × 4 every 3 weeks → CMF × 3; Arm 3 (sequential docetaxel): (A-T) A 75 mg/m^2^ × 3 every 3 weeks→ docetaxel (T) 100 mg/m^2^ × 3 every 3 weeks → CMF × 3; Arm 4 (concurrent docetaxel): (AT) AT 50/75 mg/m^2^ × 4 every 3 weeks → CMF × 3. Full details and a CONSORT diagram were previously reported^36^. Patients were followed up to 10 years from recruitment of the last patient. During the follow-up period, investigators were required to take patient history, perform physical examinations, and record adverse events. The study was approved by the ethics committees of all participating sites (coordinated at Institut Jules Bordet), and this sub-study was approved by the BIG 2-98 executive and translational committees and the institute where the IHC staining was carried out (HMO 14-0366).

### Central pathology review and TMA construction

A primary tumor sample (blocks or slides) was required for central pathology review. Primary tumor samples were stored centrally at the Institut Jules Bordet, Brussels, Belgium. Slide reviews were carried out on whole tissue sections from formalin-fixed paraffin-embedded (FFPE) samples at the European Institute of Oncology, Milan, Italy. Immunostaining experiments for the localization of ER and PgR as well as HER2 protein were carried out on consecutive tissue sections by means of an automated immunostainer (Autostainer, Dako, Glostrup, Denmark). The following primary antibodies were used: the 1D5 monoclonal antibody (mAb) to ER (Dako, at 1/100 dilution), the 1A6 mAb to PgR (Dako, 1/800), and the polyclonal antiserum (Dako, 1/800) to the HER2 protein. Only nuclear reactivity was taken into account for ER and PgR, and the results were recorded as the percentage of immunoreactive cells over at least 2000 neoplastic cells. FISH was carried out for HER2 according to the manufacturer’s instructions (Vysis-Abbott). Positivity thresholds were ER ≥ 1%, PgR ≥1%, HER2 = 3+ (>10% invasive tumor cells with intense and circumferential membrane staining) and/or FISH-positive (HER2:CEP17 ratio ≥ 2).

### Heparanase staining

The institutional review boards and the steering committee approved the biomarker protocol for the evaluation of heparanase in association with clinical outcome. From 2887 patients randomized in the BIG 2-98 trial, 2173 cases had tumor blocks that were centrally evaluated (Supplementary Fig. S2 CONSORT diagram). Tissue microarray (TMA) was constructed from 950 blocks. To ensure the highest possible reliability and reproducibility of the FFPE assessments, the following were strongly advised and employed as part of the pre-analytic processing conditions: (1) That surgical specimens receive fresh in the pathology laboratory were promptly examined and sampled. (2) That adequate dissection of the specimen is carried out before fixation. (3) That aqueous solution of formaldehyde 4% (10% formalin) isotonic and neutral is recommended for fixation. (4) That at least one section of the primary tumor is fixed in a large volume of formalin for at least 24 h before processing.

Paraffin blocks were submitted to the coordinating center. Four cores from each tumor were collected and placed in two different TMAs, with each TMA containing two cores of the same tumor. The BIG 2-98 TMA set contained 19 slides with approximately 170 tissue cores per slide. Two slides containing ER-negative samples were of low quality, and although they were stained, they could not be annotated. In total, 641 ER+ samples were interpretable for heparanase by IHC.

For IHC, the tissue microarray sections slides were deparaffinized with xylene and hydrated through graded ethanol. Heparanase was stained with anti-heparanase monoclonal antibody (ImClone Systems Inc., New York, NY) and diluted 1:400. The sections were then incubated with a conjugated horseradish peroxidase secondary Ab (anti-mouse [Histifine; Nichirei, Osaka, Japan]) for 30 min and developed with DAB. Staining with H&E and Masson trichrome staining was performed according to accepted protocols.

The extent of IHC heparanase staining was determined and scored separately for each spot and specimen by an expert breast cancer pathologist (R.S.) who was blinded to the pathologic clinical data. The staining was analyzed according to intensity (range: 0–3). A score of “not applicable” (N/A) was assigned to specimens that were uninterpretable. To define tumors as being hepranase-positive, a cut-off point of ≥2 was chosen since it was difficult to distinguish between background and a low intensity (1+) score.

Tissue microarray construction, determination of proteomic status, patient selection, assay performance, and data analysis were reported according to the Recommendations for Tumor Marker Prognostic Studies (REMARK) criteria^37^.

### Statistical analysis

Forty-two gene-expression data sets of expression profiles from more than 10,000 tumors were retrieved from public databases or authors’ websites (previously described in refs. ^38–43^) using the MetaGxBreast R package^44^. We performed a 0.95 quantile normalization in order to ensure comparability of expression values across multiple data sets. Differences in expression of heparanase according to subtype were examined using the Kruskal–Wallis test. Patients were assigned to the main breast cancer molecular subtypes using the PAM50 classification model. This was done with the genefu R package (v4.02) statistical suite^45^.

Distant metastasis-free survival was the primary survival endpoint, which was defined as the time elapsing between breast cancer diagnosis and the date of systemic relapse. When distant metastasis-free survival data were not reported, relapse-free survival information was used if available. Survival plots according to the heparanase median were drawn with the Kaplan–Meier method, and the significance of the survival differences was evaluated using the log-rank test. Interaction effects between treatment type and HPSE expression were displayed using forest plots.

For the BIG 2-98 outcome analysis, the patients were classified according to the presence of heparanase. The primary outcomes were disease-free survival (DFS) and overall survival (OS). DFS was defined as the interval between the date of randomization to the date of local, regional or metastatic relapse or second primary cancer or death from any cause. OS was calculated from the date of randomization to last follow-up or death from any cause. The chi-square test for categorical data and the unpaired Student’s *t*-test for continuous variables were used in order to determine an association between heparanase and pathologic clinical parameters. *p-*values < 0.05 were considered significant.

### Cells

Human‐heparanase (HPSE-high) and mock‐transfected (HPSE-low) MCF7 human breast carcinoma cells are available at our labxx. MCF7 and MDA-MB-231 cells were grown in DMEM medium. SKBR3 cells were grown in RPMI. All mediums were supplemented with 1 mM glutamine, 50 μg/ml streptomycin, 50 U/ml penicillin, and 10% fetal calf serum (FCS) (Biological Industries, Beit-Haemek, Israel), and cells were grown at 37 °C and 5% CO_2_. During chemotherapy, the cells maintained in appropriate medium with 1% fetal bovine serum.

### Reagents and drugs

Anti-LC3 (1:100) and anti-actin (1:500) monoclonal antibodies were purchased from Sigma. Anti Bcl-2 (1:100), and anti PARP (1:100) were purchased from Cell Signaling Technologies. Anti-heparanase monoclonal antibody 01385-126, recognizing both the 50-kDa subunit and the 65-kDa proheparanase, was kindly provided by Dr. P. Kussie (ImClone Systems) (1:400). The heparanase inhibitors, PG545 (Pixatimod) (10 μg/ml) and SST0001 (Roneparstat) (10 μg/ml), were kindly provided by the lab of Israel Vlodavsky and diluted to various concentrations with phosphate-buffered saline (PBS) prior to the assays. As a vehicle control cells were treated with PBS only. The following drugs were tested: cisplatin (10 μg/ml), Taxol (20 ng/ml), cyclophosphamide (10 μg/ml), docetaxel (10 μg/ml), 5-fluorouracil (5-FU) (20 μg/ml), doxorubicin (100 ng/ml) and tamoxifen (5–10 μM). All chemotherapy drugs were obtained from the Oncology Department, Hadassah Medical Center (Jerusalem, Israel) and diluted to various concentrations with serum-free medium prior to the assays. Tamoxifen was purchased from Sigma.

### Cell transfection

MCF7 cells (known to express low levels of endogenous heparanase^12^) were transfected with either human heparanase cDNA subcloned into the expression plasmid pCDNA3 (HPSE-high MCF7) or with a control pCDNA3 vector (HPSE-low MCF7), as previously described^21^. Stable transfected cells HPSE-high and HPSE-low were selected with G418 (800 μg/ml). To rule out the possibility of insertional mutagenesis, all the experiments involving transfected cells were conducted by means of a pooled population of HPSE-high and HPSE-low clones, each containing over 100 clones mixed together. Expression of heparanase was evaluated by real-time PCR and verified by measurements of enzymatic activity, as described below and in several earlier reports^12,21,46^.

### Heparanase activity assay

Measurements of heparanase enzymatic activity were performed as in^12,46^. Briefly, equal protein aliquots of cell lysates were incubated with the sulfate-labeled ECM for 16 h (37 °C, pH 6.2) and the supernatants containing 35S-labeled heparan sulfate degradation fragments were analyzed by gel filtration on a Sepharose CL-6B column (0.9 × 30 cm). Fractions (0.2 mL) were eluted with PBS at a flow rate of 5 ml/h and counted for radioactivity. The excluded volume (Vo) was marked by blue dextran, and the total included volume (Vt) was marked by phenol red. Nearly intact HSPGs are eluted from Sepharose 6B just after the void volume (Kav < 0.2, fractions 1–10), while HS degradation fragments are eluted toward the Vt of the column (peak II, 0.5 < Kav < 0.8, fractions 15–35). Each experiment was performed at least three times and the variation in elution positions (Kav values) did not exceed 15%. Labeled fragments eluted in peak II were shown to be degradation products of HS as they were 5–6-fold smaller than intact HS chains of HSPGs, resistant to further digestion with papain and chondroitinase ABC, and susceptible to deamination by nitrous acid^46^. Heparanase activity = Kav × total cpm in peak II.

### MTT [3-(4,5-dimethylthiazol-2-yl)]-2,5-diphenyltetrazolium bromide assay

The viability of cells was determined by the CellTiter96 nonradioactive cell proliferation kit (Promega Corp., Madison, WI). Briefly, cells were seeded at 4 × 10^3^ cells/well in 96-well microtiter plates in the appropriate medium with 1% fetal bovine serum. The cells were incubated overnight for attachment. Then, the indicated concentrations of drugs were added in triplicates, and cell viability was measured after 48 h (MDA-MB-231 and SKBR3) or 72 h (MCF7) of treatment by MTT assay according to the manufacturer´s recommendations. Experiments were repeated at least three times, and data are represented as means ± SEM.

### RNA extraction and real-time qPCR

Total RNA was extracted using a Direct-zol™ RNA MiniPrep kit (Zymo Research) from MCF7 cells using a Direct-zol™ RNA MiniPrep kit (Zymo Research) and real-time PCR were performed as described before^47^. Briefly, complementary DNA was obtained by reverse transcription of 850 ng of total RNA using Quantabio kit according to the manufacturer’s instructions. PCR was carried out using PerfeCTa SYBR Green FastMix, ROX (Quantabio). Primers (Supplementary Table 1) and probe mix for HPSE, Oct3/4, Nanog, Sox2, Snail, and Bcl-2 were purchased from Biosearch Technologies and utilized according to the manufacturer’s instructions. All reactions were run in triplicate, and the housekeeping gene, *GAPDH*, was amplified in a parallel reaction for normalization.

### RNA-seq analysis

Poly(A)-selected RNA was sequenced using the Illumina TruSeq protocol on the HiSeq 2500 sequencing machine. Quality control, read mapping, and differential expression analysis were performed as described before^28^ with the following changes. Clean reads were mapped to the human genome (hg38) using HISAT2^48^. Next, the number of reads mapping each human gene (as annotated in the gencode v29 annotation) was counted with the featureCounts program^49^. Genes with a FDR < 0.05 and a fold-change > 2 were considered as being differentially expressed. Gene set enrichment and pathway analysis were done with the GAGE R package^50^ and GeneAnalytics^51^, GSEA enrichment plot was generated using fgsea R package^52^. The RNA-sequencing data is available at Data BioProject ID: PRJNA721806.

### Flow cytometry

MCF7 cells were incubated overnight for attachment in DMEM with 1% fetal bovine serum. The cells were then treated with 5-FU (20 μg/ml) for 72 h, after which the medium was replaced and the cells were incubated in DMEM with 10% fetal bovine serum for an additional 72 h and detached from the cell culture plates by using Accutase. The cells were stained with Brilliant Violet 421™ anti-human CD24 antibody, BioLegend, and Brilliant Violet 510™ anti-mouse/human CD44.

### Reporting summary

Further information on research design is available in the Nature Research Reporting Summary linked to this article.

## Supplementary information

Supplementary Information

Reporting Summary

## Data Availability

The data generated and analyzed during this study are described in the following data record: 10.6084/m9.figshare.14485065^53^. The RNA-sequencing data are openly available in the Sequence Read Archive via the following accession: https://identifiers.org/ncbi/bioproject:PRJNA721806^54^. The immunostaining data and FACS raw data files (fcs) are stored on a hard disc in the corresponding author’s lab (file name: Heparanase BIG 2-98). These files are available upon request to the corresponding author. The study clinical/pathological data and IHC staining data of heparanase are available upon request and located at the BIG institutional storage as well as personal hard drive at the corresponding author’s lab.
