# Supplementary material for: Heparanase: a potential marker of worse prognosis in estrogen receptor-positive breast cancer
Source: NPJ Breast Cancer. 2021 May 28;7:67. doi: 10.1038/s41523-021-00277-x (PMC8163849; doi:10.1038/s41523-021-00277-x)
Supplement: Supplementary file 1 — Supplementary Information [file 41523_2021_277_MOESM1_ESM.pdf]

Supplementary information

Supplementary method

Methylene blue assay

Methylene blue assay was performed as described elsewhere[53]. Briefly, cells were fixed in glutaraldehyde at a final concentration of 0.05% and were stained with 1% methylene blue in 0.1 M borate buffer, pH 8.5. The dye taken up by cells was eluted in 0.1 N HCl for 60 min at 37 °C, and absorbance was monitored at 620 nm. Each point of the growth curve experiments was calculated from 5 wells.

Supplementary Figure 1

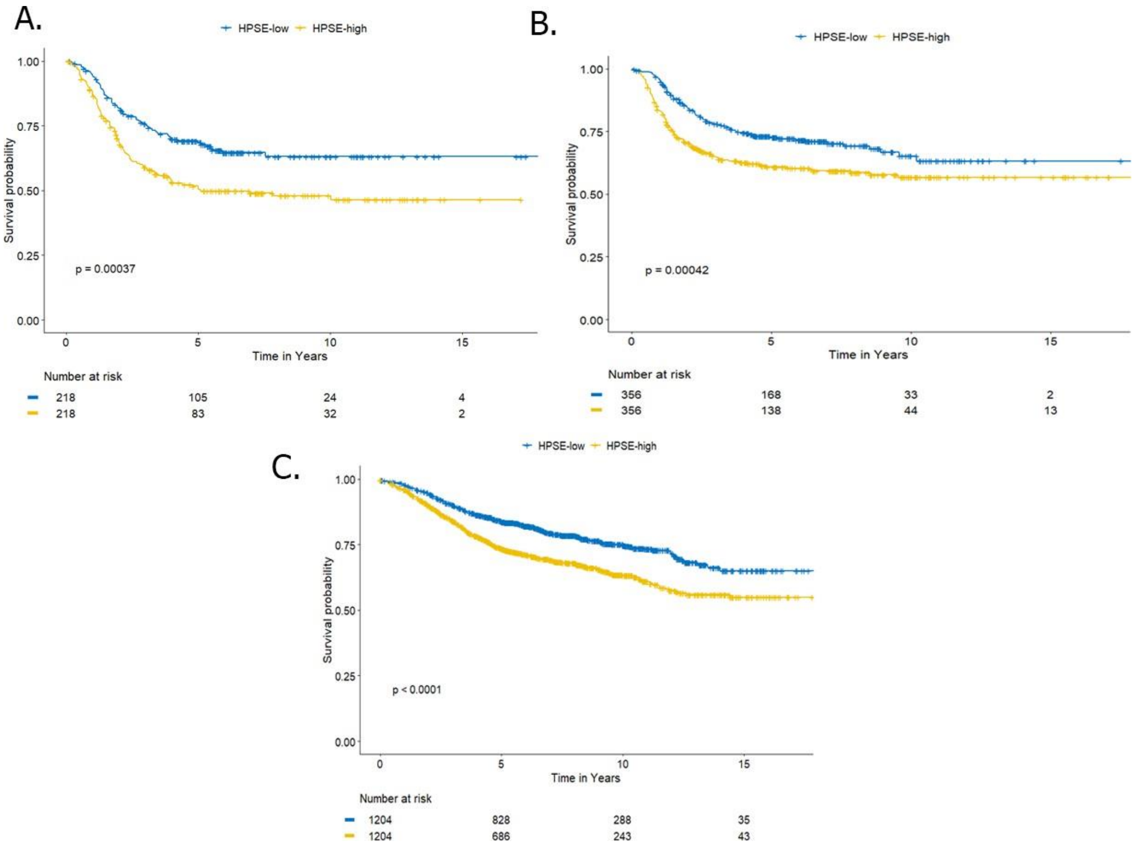

Supplementary Figure 1. (A-C) We assessed the prognostic value of quartiles of heparanase gene expression in HER2+ (A), Basal (B) and Luminal (C) breast cancer patients. Significance (p values) of differences in survival between patient groups as defined by quartiles of heparanase expression is estimated by the log-rank test.

Supplementary Figure 2

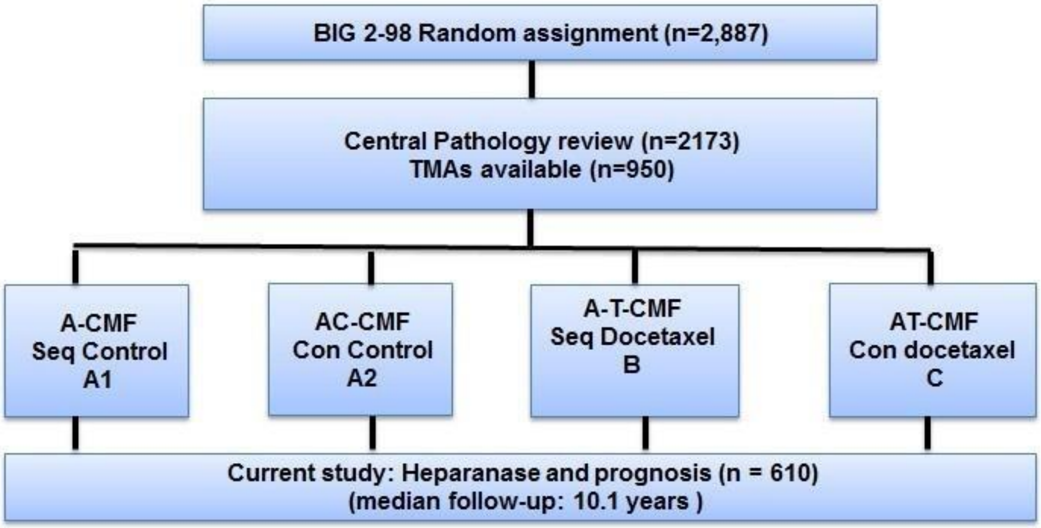

Supplementary Figure 2. CONSORT diagram of the BIG 2-98. Arm A1 sequential control; Arm A2 concurrent control; Arm B sequential docetaxel; Arm C concurrent docetaxel.

Supplementary Figure 3

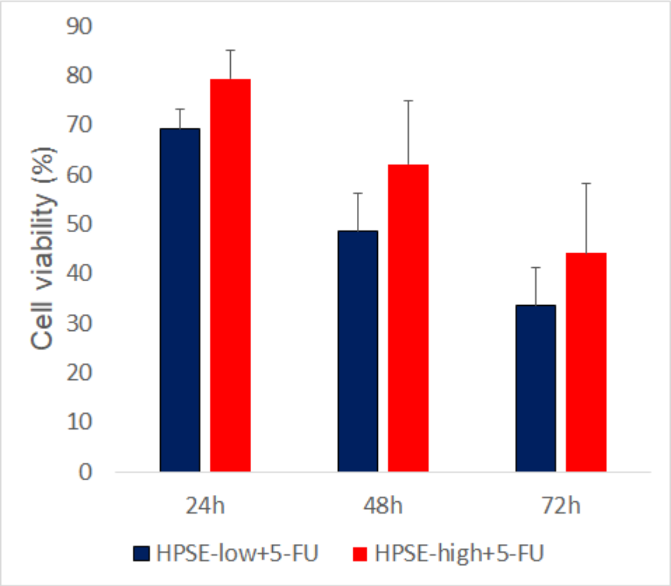

Supplementary Figure 3. Effect of 5-FU treatment on the viability of HPSE-high MCF7 cells. HPSE-low or HPSE-high cells that were treated with 5-FU chemotherapy agent for 24h, 48h and 72h. Cell viability percentage of control vs. treated cells was determined with methylene blue assay. The data are presented as mean ± SEM from three independent experiment in triplicate.

Supplementary Figure 4

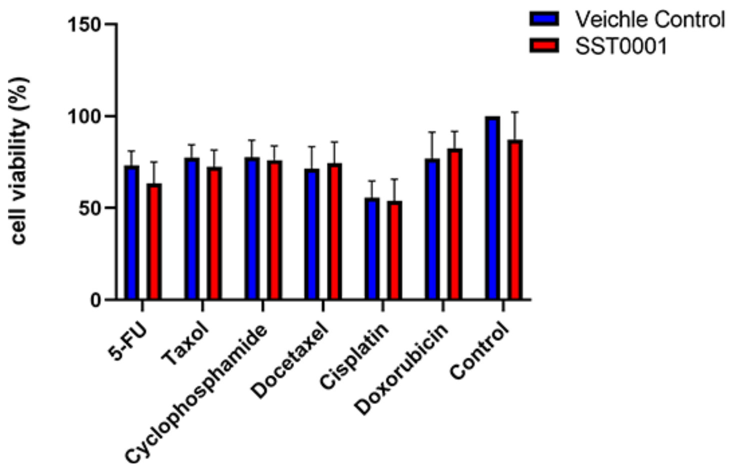

Supplementary Figure 4. MDA-MB-231 breast cancer cells were treated with SST0001 and different chemotherapy drugs for 48h after which cell viability was determined by MTT assay. No significant difference was observed following chemotherapy treatment in comparison with the combination therapy of the chemo and heparanase inhibitor.

Supplementary Figure 5

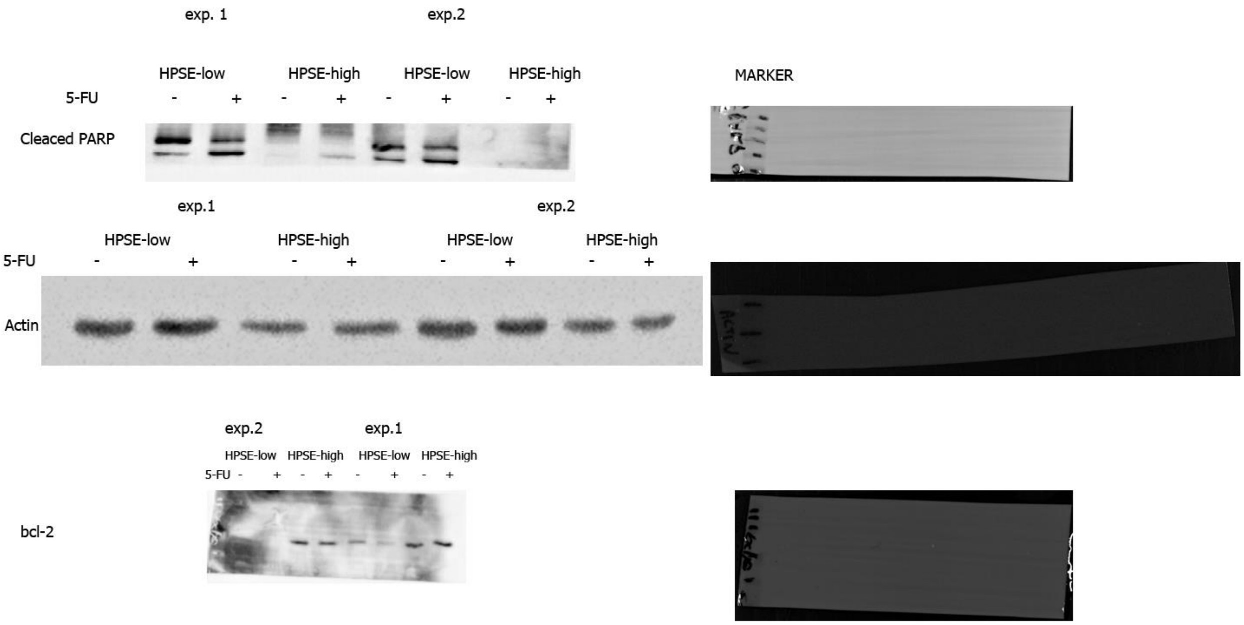

Supplementary Figure 5. Western blot analysis of Actin, Bcl-2 and cleaved PARP from HPSE-low or HPSE-high following 5-FU treatment for 24h. The original blots represent two different experiments run together. Notably, for each sample, equal volume was loaded in each of the running gel.

Supplementary Table 1. List of primers for real-time qPCR.

Gene	Primers
**GAPDH**	**F:** TCACCACCATGGAGAAGGC
	**R:** GCTAAGCAGTTGGTGGTGCA
**HPSE**	**F:** TCAAGAACAGCACCTACTCAAG
	**R:** AACGCATTTAGGCCAAAGATCA
**Snail**	**F:** ACCACTATGCCGCGCTCTT
	**R:** GGTCGTAGGGCTGCTGGAA
**Oct3/4**	**F:** AGGAGAAGCTGGAGCAAAACC
	**R:** ATCCTCTTCTGCTTCAGGAGCT
**Sox2**	**F:** GAGTGGAAACTTTTGTCGGAGAC
	**R:** GTATTTATAATCCGGGTGCTCCTTCA
**Bcl-2**	**F:** CCGTTGGCCCCCGTTGCTGT
	**R**: CTCGCGGAGGGTCAGGTGGA
**Nanog**	**F:** CTCCAACATCCTGAACCTCAGC
	**R:** CGTCACACCATTGCTATTCTTCG

F, forward; R, reverse.
