# Supplementary material for: Heparanase: a potential marker of worse prognosis in estrogen receptor-positive breast cancer
Source: NPJ Breast Cancer. 2021 May 28;7:67. doi: 10.1038/s41523-021-00277-x (PMC8163849; doi:10.1038/s41523-021-00277-x)
Supplement: Supplementary file 2 — Reporting Summary [file 41523_2021_277_MOESM2_ESM.pdf]

nature research

Corresponding author(s): Amir Sonnenblick

Last updated by author(s): Feb 19, 2021

Reporting Summary

Nature Research wishes to improve the reproducibility of the work that we publish. This form provides structure for consistency and transparency in reporting. For further information on Nature Research policies, see our Editorial Policies and the Editorial Policy Checklist.

Statistics

For all statistical analyses, confirm that the following items are present in the figure legend, table legend, main text, or Methods section.

n/a	Confirmed	
☐	☒	The exact sample size (*n*) for each experimental group/condition, given as a discrete number and unit of measurement
☐	☒	A statement on whether measurements were taken from distinct samples or whether the same sample was measured repeatedly
☐	☒	The statistical test(s) used AND whether they are one- or two-sided<br>*Only common tests should be described solely by name; describe more complex techniques in the Methods section.*
☐	☒	A description of all covariates tested
☐	☒	A description of any assumptions or corrections, such as tests of normality and adjustment for multiple comparisons
☐	☒	A full description of the statistical parameters including central tendency (e.g. means) or other basic estimates (e.g. regression coefficient) AND variation (e.g. standard deviation) or associated estimates of uncertainty (e.g. confidence intervals)
☒	☐	For null hypothesis testing, the test statistic (e.g. *F*, *t*, *r*) with confidence intervals, effect sizes, degrees of freedom and *P* value noted<br>*Give P values as exact values whenever suitable.*
☒	☐	For Bayesian analysis, information on the choice of priors and Markov chain Monte Carlo settings
☐	☒	For hierarchical and complex designs, identification of the appropriate level for tests and full reporting of outcomes
☒	☐	Estimates of effect sizes (e.g. Cohen's *d*, Pearson's *r*), indicating how they were calculated

Our web collection on statistics for biologists contains articles on many of the points above.

Software and code

Policy information about availability of computer code

Data collection	Forty-two gene expression datasets of expression profiles from more than 10,000 tumors were retrieved from public databases or authors' websites (previously described in ref 42) using the MetaGxBreast R package (ref43).
Data analysis	Genes with a FDR < 0.05 and a fold-change > 2 were considered as being<br>differentially expressed. Gene set enrichment and pathway analysis were done with the GAGE R package ref50 and GeneAnalytics ref51, GSEA enrichment plot was generated using fgsea R package ref52.

For manuscripts utilizing custom algorithms or software that are central to the research but not yet described in published literature, software must be made available to editors and reviewers. We strongly encourage code deposition in a community repository (e.g. GitHub). See the Nature Research guidelines for submitting code & software for further information.

Data

Policy information about availability of data

All manuscripts must include a data availability statement. This statement should provide the following information, where applicable:

- Accession codes, unique identifiers, or web links for publicly available datasets
- A list of figures that have associated raw data
- A description of any restrictions on data availability

The datasets generated during and/or analysed during the current study are available from the corresponding author on reasonable request

Field-specific reporting

Please select the one below that is the best fit for your research. If you are not sure, read the appropriate sections before making your selection.

☒ Life sciences ☐ Behavioural & social sciences ☐ Ecological, evolutionary & environmental sciences

For a reference copy of the document with all sections, see nature.com/documents/nr-reporting-summary-flat.pdf

Life sciences study design

All studies must disclose on these points even when the disclosure is negative.

Sample size	From 2,887 patients randomized in the BIG 2-98 trial, 2,173 cases had tumor blocks that were centrally evaluated (Fig. S2 CONSORT diagram). Tissue microarray (TMA) was constructed from 950 blocks.
Data exclusions	Tissue microarray (TMA) was constructed from 950 blocks.
Replication	*Describe the measures taken to verify the reproducibility of the experimental findings. If all attempts at replication were successful, confirm this OR if there are any findings that were not replicated or cannot be reproduced, note this and describe why.*
Randomization	Four cores from each tumor were collected and placed in two different TMAs, with each TMA containing two cores of the same tumor.
Blinding	The extent of IHC heparanase staining was determined and scored separately for each spot and specimen by an expert breast cancer pathologist (R.S.) who was blinded to the pathologic clinical data.

Reporting for specific materials, systems and methods

We require information from authors about some types of materials, experimental systems and methods used in many studies. Here, indicate whether each material, system or method listed is relevant to your study. If you are not sure if a list item applies to your research, read the appropriate section before selecting a response.

Materials & experimental systems

n/a	Involved in the study	
☐	☒	Antibodies
☐	☒	Eukaryotic cell lines
☒	☐	Palaeontology and archaeology
☒	☐	Animals and other organisms
☐	☒	Human research participants
☐	☒	Clinical data
☒	☐	Dual use research of concern

Methods

n/a	Involved in the study	
☒	☐	ChIP-seq
☐	☒	Flow cytometry
☒	☐	MRI-based neuroimaging

Antibodies

Antibodies used	Anti-LC3 (1:100) and anti-actin (1:500) monoclonal antibodies were purchased from Sigma. Anti Bcl-2 (1:100), and anti PARP (1:100) was purchased from Cell Signaling Technologies. Anti-heparanase monoclonal antibody 01385–126, recognizing both the 50-kDa subunit and the 65-kDa proheparanase, was kindly provided by Dr. P. Kussie (ImClone Systems) (1:400).
Validation	Expression of heparanase was evaluated by RT-PCR and verified by measurements of enzymatic activity, as described below and in several earlier reports

Eukaryotic cell lines

Policy information about cell lines

Cell line source(s)	MCF7 cells (known to express low levels of endogenous heparanase12) were transfected with either human heparanase cDNA subcloned into the expression plasmid pCDNA3 (HPSE-high MCF7) or with a control pCDNA3 vector (HPSE-low MCF7), as previously described21.
Authentication	All cell lines were authenticated

Mycoplasma contamination	cell lines were not tested for mycoplasma contamintaion
Commonly misidentified lines (See ICLAC register)	N/A

Human research participants

Policy information about studies involving human research participants

Population characteristics	BIG 2-98 (ClinicalTrials.gov identifier of BIG 2-98: NCT00174655) is a multicenter, prospective, open-labelled, randomized phase III adjuvant trial that enrolled early and locally advanced lymph node positive breast cancer patients who were assigned to different adjuvant chemotherapy regimens.
Recruitment	Full details and a CONSORT diagram were previously reported ref40.
Ethics oversight	The institutional review boards and the steering committee approved the biomarker protocol for the evaluation of heparanase in association with clinical outcome. (HMO 14-0366)

Note that full information on the approval of the study protocol must also be provided in the manuscript.

Clinical data

Policy information about clinical studies

All manuscripts should comply with the ICMJE guidelines for publication of clinical research and a completed CONSORT checklist must be included with all submissions.

Clinical trial registration	ClinicalTrials.gov identifier of BIG 2-98: NCT00174655
Study protocol	randomized phase III adjuvant trial ref 22
Data collection	From 2,887 patients randomized in the BIG 2-98 trial, 2,173 cases had tumor blocks that were centrally evaluated (Fig. S2 CONSORT diagram)
Outcomes	Heparanase Kaplan-Meier survival curves in the BIG 2-98 cohort.

Flow Cytometry

Plots

Confirm that:

☒ The axis labels state the marker and fluorochrome used (e.g. CD4-FITC).

☒ The axis scales are clearly visible. Include numbers along axes only for bottom left plot of group (a 'group' is an analysis of identical markers).

☒ All plots are contour plots with outliers or pseudocolor plots.

☒ A numerical value for number of cells or percentage (with statistics) is provided.

Methodology

Sample preparation	MCF7 cells were incubated overnight for attachment in DMEM with 1% fetal bovine serum. The cells were then treated with 5-FU (20ug/ml) for 72h, after which the medium was replaced and the cells were incubated in DMEM with 10% fetal bovine serum for an additional 72h and detached from the cell culture plates by using Accutase
Instrument	The provided results are from a representative experiment repeated three times
Software	As above
Cell population abundance	No sorting
Gating strategy	The cells were stained with Brilliant Violet 421™ anti-human CD24 antibody, BioLegend, and Brilliant Violet 510™ anti-mouse/human CD44.

☒ Tick this box to confirm that a figure exemplifying the gating strategy is provided in the Supplementary Information.
